# Effects of white matter hyperintensities distribution and clustering on late-life cognitive impairment

**DOI:** 10.1038/s41598-022-06019-8

**Published:** 2022-02-04

**Authors:** Joan Jiménez-Balado, Fabian Corlier, Christian Habeck, Yaakov Stern, Teal Eich

**Affiliations:** 1 Leonard Davis School of Gerontology, University of Southern California, Los Angeles, CA USA; 2 Department of Neurology, Taub Institute for Research on Alzheimer’s Disease and the Aging Brain, Vagelos College of Physicians and Surgeons, Columbia University, New York, NY USA

**Keywords:** Neuroscience, Biomarkers, Diseases, Neurology

## Abstract

White matter hyperintensities (WMH) are a key hallmark of subclinical cerebrovascular disease and are known to impair cognition. Here, we parcellated WMH using a novel system that segments WMH based on both lobar regions and distance from the ventricles, dividing the brain into a coordinate system composed of 36 distinct parcels (‘bullseye’ parcellation), and then investigated the effect of distribution on cognition using two different analytic approaches. Data from a well characterized sample of healthy older adults (58 to 84 years) who were free of dementia were included. Cognition was evaluated using 12 computerized tasks, factored onto 4 indices representing episodic memory, speed of processing, fluid reasoning and vocabulary. We first assessed the distribution of WMH according to the bullseye parcellation and tested the relationship between WMH parcellations and performance across the four cognitive domains. Then, we used a data-driven approach to derive latent variables within the WMH distribution, and tested the relation between these latent components and cognitive function. We observed that different, well-defined cognitive constructs mapped to specific WMH distributions. Speed of processing was correlated with WMH in the frontal lobe, while in the case of episodic memory, the relationship was more ubiquitous, involving most of the parcellations. A principal components analysis revealed that the 36 bullseye regions factored onto 3 latent components representing the natural aggrupation of WMH: fronto-parietal periventricular (WMH principally in the frontal and parietal lobes and basal ganglia, especially in the periventricular region); occipital; and temporal and juxtacortical WMH (involving WMH in the temporal lobe, and at the juxtacortical region from frontal and parietal lobes). We found that fronto-parietal periventricular and temporal & juxtacortical WMH were independently associated with speed of processing and episodic memory, respectively. These results indicate that different cognitive impairment phenotypes might present with specific WMH distributions. Additionally, our study encourages future research to consider WMH classifications using parcellations systems other than periventricular and deep localizations.

## Introduction

White matter hyperintensities (WMH) of presumed vascular origin are a common finding in healthy older adults, with prevalence rates ranging between 39 and 96%^1–3^. Beyond age, the most important contributor to WMH progression is sustained exposure to vascular risk factors including hypertension^4^, chronic kidney disease^5^, diabetes, and dyslipidemia^6^. These risk factors cause significant structural changes in the small vessels of the brain including narrowing of the lumen and loss of smooth muscle cells, which ultimately result in impaired vascular tone, endothelial damage, and blood–brain barrier breakdown^7–9^. After the onset of these mechanisms, WMH accumulates on brain parenchyma at a rate ranging from 0.1 to 2.2 mL/year^10^. Although this progression is subclinical, it increases the likelihood of incident mild cognitive impairment (MCI)^11^, dementia, stroke^12^ and gait disturbances^13^.

The regional distribution of WMH has been shown to contribute to the course of clinical symptoms^14^. When classified according to the deep subcortical (D-WMH) vs periventricular region (PV-WMH) distribution proposed by Fazekas and colleagues (1987)^15^, several studies revealed that both burden and progression of PV-WMH were more strongly related to cognitive decline and incident MCI than were D-WMH burden and progression^11,16,17^. PV-WMH have also been associated with reduced cortical thickness and volume^18,19^. Additionally, there is also evidence that lobar anatomy might influence the effect of WMH on cognitive impairment: a previous study by Moura et al. (2019) that assessed the relation between cognition and WMH according to the lobar region found that parietal WMH mediated the relation between age and performance on the speed of processing tasks^20^. While the D/PV- distribution of WMH is commonly used, some researchers have challenged this distinction. DeCarli and colleagues (2005), for example, argued that the qualitative assessment of PV-WMH and D-WMH is controversial, as it is based on the distance from the ventricles using arbitrary cutoffs without any consensus and confounded by the evaluation of WMH in the axial plane^21^. On the other hand, lobar parcellations do not consider the distance from the ventricles and, if we consider WMH to be a dynamic disorder in which there is an evolution from the ventricles to the brain parenchyma^14^, this might be a relevant parameter as an indicator of the stage of WMH progression. Hence, traditional classifications of WMH may fail to capture the true and more subtle nature of WMH spatial distribution.

Sudre and collaborators (2018) recently proposed a novel classification of WMH based on both lobar regions and distance from the ventricles, dividing the brain into a coordinate system composed of 36 distinct parcels, which they refer to as a ‘bullseye’ parcellation^22^. This parcellation of brain white matter was originally designed to train raters in the administration of WMH visual scales, which could then be correlated with WMH volume in different brain regions^22^. However, the bullseye parcellation has since been used to investigate the relation between specific patterns of WMH and profiles of risk factors^23^ and dementia presentations^24^. The results of these studies underscore the idea that WMH distribution may be influenced by patients’ phenotypes, and that the bullseye classification system of WMH represents an important tool that can be used to objectively capture these differences. Further, the bullseye classification system may also provide a means to gain insight into the natural categorization of WMH beyond PV- and D-WMH^25^. This could generate new knowledge about the typology of WMH within the brain parenchyma and help to determine which patterns or distributions pose a greater risk of cognitive impairment.

The current study was thus designed to with four goals in mind. First, we used data from a large sample of cognitively healthy older adults to evaluate WMH distribution according to the bullseye parcellation. Second, we tested the relationship between this more refined localization of WMH and performance across four broad domains of cognitive functioning as measured by summarized performance across multiple tests of episodic memory, speed of processing, vocabulary, and reasoning. Third, we used principal component analysis (PCA) to derive latent variables within WMH distribution according to different brain regions. And fourth, for clinical utility and to provide summarized information of the independent contribution of each WMH latent component to cognitive impairment, we tested whether these latent components were associated with cognitive function.

## Methods

### Sample

Data from 108 healthy older adults (59 females, 54.6%) with an age ranging from 58 to 84 (median: 69, interquartile range: 64–73) and an average of 15 years of schooling (interquartile range: 14–18) who underwent both FLAIR and T1 MRI sequences was included (Supplementary Fig. 1 and see also Supplementary Table 1 for a complete descriptive analysis of the sample). Participants were recruited from a large, ongoing study in the Department of Neurology at Columbia University, the Reference Ability Neural Network study^26^. Sociodemographic and risk factors data was collected at the baseline visit, and all participants were required to be native English speakers, right-handed, have at least a fourth grade reading level, and be free of dementia.

### Neuroimaging

#### Acquisition

The acquisition procedures have been previously described^26^. Imaging data were acquired on 3.0 T Philips Achieva Magnet. Each participant underwent fMRI scanning while performing 12 computerized cognitive tasks, described in detail below. Tasks were administered over the course of two 2-h scanning sessions on different days, with six tasks administered in each scanning-session. At each session, a scout, T1-weighted image was acquired to determine participant position. Participants underwent a T1-weighted MPRAGE scan to determine brain structure, with a TE/TR of 3/6.5 ms and Flip Angle of 8 degrees, in-plane matrix size of 256 × 256, field of view of 256 mm × 256 mm, and 180 slices in the axial direction with a slice-thickness/gap of 1/0 mm. The FLAIR sequence had a TE/TR of 125/11,000 ms and Flip Angle of 90 degrees, in-plane matrix size of 288 × 288, field of view of 23 mm × 23 mm, and consisted of 30 slices with a slice-thickness/gap of 4/0.5 mm. A neuroradiologist reviewed each participant’s scans.

#### Cortical and subcortical segmentation

MPRAGE T1 structural 3D images were processed with Freesurfer’s pipeline (http://surfer.nmr.mgh.harvard.edu). Images were first preprocessed by applying N3 bias field correction, intensity normalization and skull stripping. Normalized T1 sequences were then registered to a standard template to obtain subcortical anatomical segmentations, and tissue class segmentation was used to obtain the white matter mask. Finally, tessellation was used to calculate the boundaries separating gray and white matter, as well as the pial surface. Spherical registration was used to align subjects’ cortical surfaces, which were mapped according to Desikan-Killiany atlas^27–29^. All outputs were visually inspected and manually corrected when necessary, following the Freesurfer’s manual editing guidelines.

Brain parenchymal fraction (BPF) was calculated using the supratentorial brain volume (gray and white matter) and the estimated total intracranial volume (eTIV), obtained via the transform matrix from native space to MNI-305 space according to the following formula^30^:

$$BPF=\frac{Supratentorial\,\, Brain\,\, Volume}{eTIV}\cdot 100$$

#### WMH segmentation

Total WMH burden was quantified from FLAIR images using BIANCA (Brain Intensity AbNormality Classification Algorithm), a fully-automated program, implemented in FSL (https://fsl.fmrib.ox.ac.uk/fsl)^31–33^ and based on the k-NN (k-nearest neighbor) algorithm. First, brain-extracted and bias-corrected FLAIR images were co-registered to the structural T1 images using Andvanced Normalization Tools (ANTs)^34,35^. Then, image intensities were standardized into Z-score using Neurobase library, implemented in R^31,36^. The BIANCA program was trained with manual WMH segmentations from 60 participants including intensity features of T2-FLAIR and T1 images, as well as the transformation matrix of each native space to MNI coordinates to account for the localization of lesions. BIANCA outputs consisted of a probability map for each participant, which represented the probability of each voxel being a lesion. After the training was completed, the performance of the BIANCA classifier in the training set was evaluated and a cutoff of probability that yielded the highest accuracy (0.9) was calculated. Finally, the BIANCA classifier was applied to the full sample to create a WMH binary mask for each participant. These masks were used to calculate the total WMH volume in mm^3^ using the ‘fslstats’ function^32^. All images were visually inspected by a trained rater and edited when required.

#### WMH parcellation

**Figure 1:**
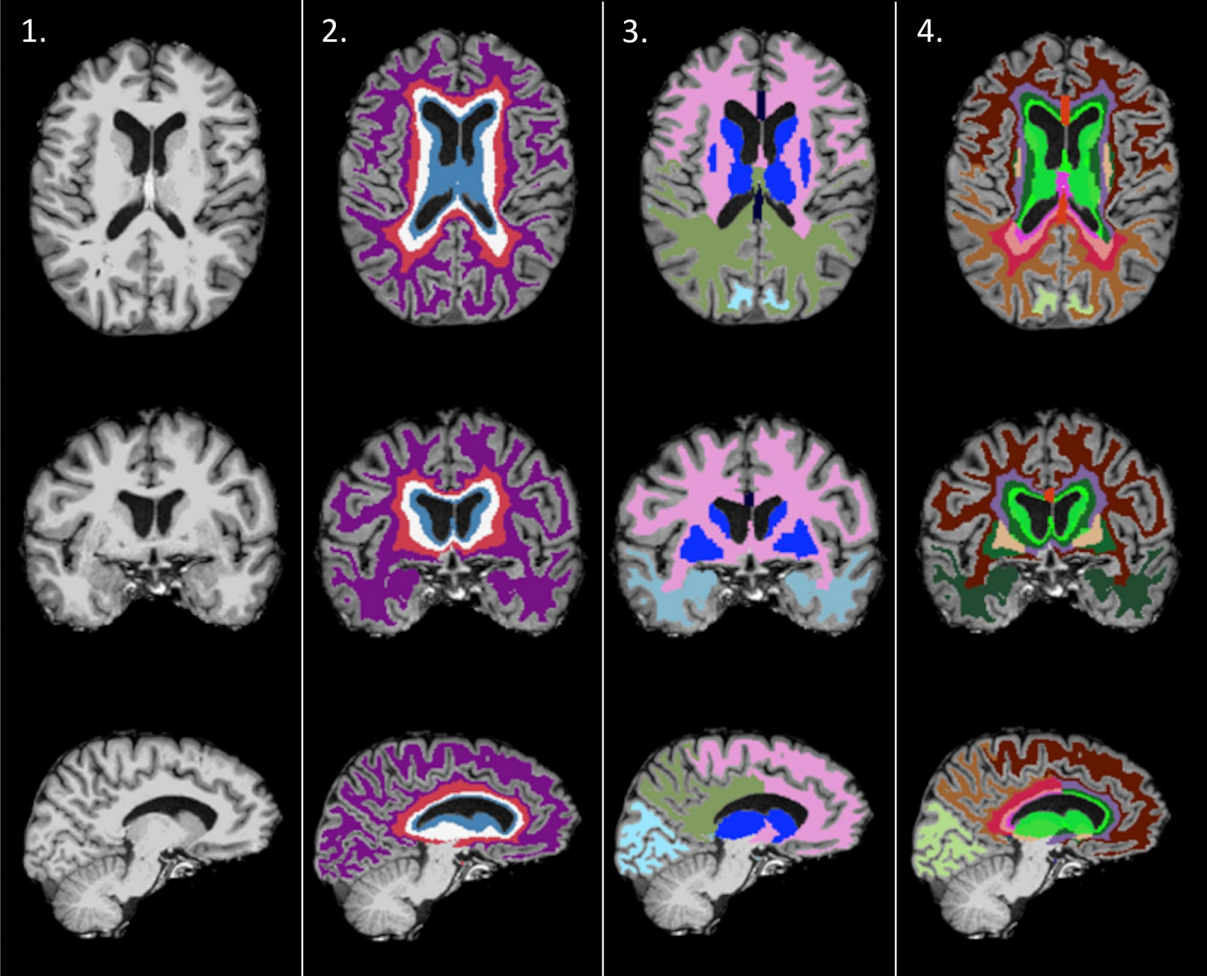
Bullseye parcellation of WMH. White matter from brain-extracted T1 images (1st column) was parcellated both in 4 equidistant layers representing the distance from the cortex to the ventricles (2nd column), and 9 regions representing either the lobe of each hemisphere or the basal ganglia (3rd column). Bullseye parcellation represents the intersection between these maps (4th column). This figure was constructed using Freesurfer software (version 7.1., https://surfer.nmr.mgh.harvard.edu/) and GNU image manipulation software (GIMP, version 2.10.22, https://www.gimp.org/).

WMH segmentation was parcellated following the bullseye classification system using a custom in-house script written mostly in Python^22^. First, we created two sets of parcellations: concentric layers and lobar regions. For concentric layers, we created two masks: one representing the distance from each voxel to the cortex, and the other representing the distance from each voxel to the ventricles. A normalized distance map was then obtained using the following formula for each voxel:

$$\frac{{Distance\,\, to\,\, ventricles}_{i}}{\left({Distance\,\, to\,\, ventricles}_{i}+ {Distance\,\, to\,\, cortex}_{i}\right)}$$

where ‘i’ refers to each voxel. This map represents the distance from each voxel to the lateral-ventricles, where ‘0’ refers to the lateral-ventricles and ‘1’ to cortex segmentations. This normalized distance was then divided in four concentric equidistant layers representing the following distances: [0, 0.25), [0.25, 0.5), [0.5, 0.75), [0.75, 1] (see Fig. 1, 2nd column). These layers were named as 1 to 4, and they extend from the ventricles to the juxtacortical location. For lobar regions, white matter was labeled according to frontal, parietal, temporal, and occipital lobe parcellations obtained with Freesurfer’s pipeline, which were projected onto the white matter using the ‘mri_aparc2aseg’ function (Fig. 1, 3rd column). The Basal Ganglia (BG) was labeled considering caudate, putamen, thalamus and globus pallidus subcortical segmentations. Finally, the bullseye parcellation was created as the intersection of both the concentric and lobar maps (Fig. 1, 4th column), resulting in 36 parcels (9 lobar regions with 4 layers each). Therefore, the number of voxels in the 36 bullseye regions is equivalent to the original number of voxels located at the white matter and basal ganglia. All maps were visually inspected.

WMH parcellation was adjusted by the eTIV using the following formula:

$$Adjusted \,\,WM{H}_{i,j}=WM{H}_{i,j}\cdot \frac{eTI{V}_{mean}}{eTI{V}_{i}}$$

where ‘i’ and ‘j’ represent the patient and parcellation, respectively.

### Cognitive protocol

Participants completed a cognitive battery consisting of 12 tasks including 3 tasks from each of the following four cognitive domains: Episodic Memory, Fluid Reasoning, Speed of Processing and Vocabulary. Episodic memory scores were measured as the proportion of correctly answered questions from the Logical memory (the number of idea units recognized across three stories), Free recall (the number of words recognized across four trials of a word list) and Paired associates (recognition of the second words from word pairs) tasks. Speed of Processing was measured using reverse-scored reaction times for correct trials on the following three tasks: Digit symbol (use a code table to select the correct symbol for a digit), Letter comparison (indicate whether pairs of letter strings are same or different) and Pattern comparison (indicate whether pairs of pairs of line patterns are same or different). Fluid reasoning was assessed with the proportion of correct trials from the Paper Folding (select the pattern of holes that would result from a sequence of folds and a punch through the folded paper^37^), Matrix Reasoning (select which pattern best completes the missing cell in a matrix^38^) and Letter Sets (select which of five groups of letters is different from the others^37^) tasks. Vocabulary measures were the proportion of correct responses for the Synonyms (select the best synonym of the target word), Antonyms (select the best antonym of the target word^39^) and Picture Naming (select the beginning letter of the name of the pictured object^40^) tasks. The tasks are described in detail in Stern et al. (2014)^26^ and in Habeck et al. (2016)^41^.

As previously described^26^, in the protocol, one session presented the episodic memory and fluid reasoning tasks, interspersed in a fixed order. The other session presented the vocabulary and speed of processing tasks, also interspersed in a fixed order. While the order of tasks within session was not varied, the order of the two sessions was counterbalanced across participants. Prior to each scan session, computerized training was administered for the six tasks to be administered during that session. At the completion of training for each task, participants had the option of repeating the training. During training, responses were made on a computer keyboard. During scanning, for all tasks except for picture naming, task responses were made on a LUMItouch response system and behavioral response data were recorded on the task computer. The picture naming test utilized a verbal response recorded from an in-scanner microphone, from which behavioral performance was determined after the scan.

Task administration and data collection were controlled by a computer running EPrime software, and electronically synchronized with the MR scanner. Task stimuli were back-projected onto a screen located at the foot of the MRI bed using an LCD projector. Participants viewed the screen via a mirror system located in the head coil, and, if needed, had vision corrected to normal using MR compatible glasses (manufactured by SafeVision, LLC. Webster Groves, MO). Task onset was electronically synchronized with the MRI acquisition computer.

### Statistics

Standardized performance (mean = 0 ± 1) across the four cognitive domains (speed of processing, episodic memory, fluid reasoning and vocabulary) were considered as outcome variables, where higher scores represented better performance (speed of processing was reverse-scored). Distribution of brain-wide WMH was investigated using histograms.

To investigate the influence of age on WMH distribution, we split the sample according to age tertiles and compared WMH across groups using the general linear model assuming a gamma distribution with a log link.

To investigate the relationship between WMH load and cognitive domains, we first log transformed WMH to achieve a linear relationship between WMH and cognitive performance. Next, multiple linear regression models including performance in each cognitive domain as dependent variables and fitting one model for each WMH region were computed. All models were adjusted for age, sex, years of education BPF and hypertension (all measured by self-report), and all results were corrected for multiple testing using a false discovery rate (FDR) of 5% according to the Benjamini and Hochberg calculation^42^.

To investigate the latent structure of WMH within the bullseye representation, we conducted a PCA using the correlation matrix of these 36 log-transformed parcels and generated scores that represented the linear combination of these variables^43^. This allowed us to observe how the bullseye regions grouped according to their correlation. As we assumed that resulting scores would be correlated, oblimin (oblique) rotation was used. To extract the correct number of variables, we conducted a parallel analysis comparing scree plots of actual and simulated data^43,44^. The resulting principal components (PCs) were interpreted by observing their correlation with WMH parcellation. Further, their association with demographic variables was explored using Pearson correlation coefficients or *t*-tests, according to the type of the variable (continuous or categorical).

Finally, to investigate whether the PCA-derived latent components were associated with cognitive performance, we conducted multiple linear regression models using the four component cognitive scores as dependent variables in separate models. These models were adjusted for age, sex, education BPF and hypertension. Since oblique rotation was used in the PCA, we tested for the presence of multicollinearity in each model by assessing variance inflation factors. Other assumptions (linear association, normality of residuals, absence of influential cases) were also checked and met. Additionally, confidence intervals were bootstrapped doing 1000 resampling iterations and calculating the 2.5 and 97.5 percentiles from β coefficients’ distributions.

All statistical analyses were conducted using R (version 4.0.1, 2020-10-10; © 2019). α was set at 0.05.

### Ethics approval

The study was approved by the Columbia University Medical Center Human Subjects IRB (AAAI2752). Written informed consent was obtained prior to testing, and participants were compensated.

## Results

### Distribution of WMH in the sample

**Figure 2:**
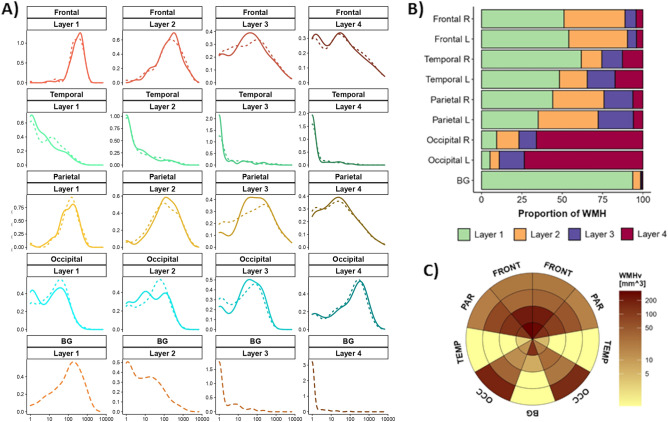
WMH distribution in the sample (N = 108). **(A)** Density plots of WMH in the sample. Y axis corresponds to the density function, while X axis corresponds to the WMH volume in mm^3^ (and scaled to log10). Colors correspond to the lobar regions (frontal, temporal, parietal, occipital lobes, and basal ganglia) and line types to the hemisphere (solid lines: left hemisphere; short-dashed lines: right hemisphere; long-dashed lines: both hemispheres, only in basal ganglia). **(B)** Proportion of WMH per layer in each lobar region. Colors correspond to the layer number (1 to 4) according to the legend. **(C)** Median WMH in each parcel of the bullseye representation in the whole sample. Colors represent the burden of WMH in mm^3^. Darker colors indicate higher WMH loads. This figure was constructed using ‘ggplot’ library (version 3.3.2, https://ggplot2.tidyverse.org/) included in R software (R version 3.6.3, 2020-02-29; 2020 The R Foundation for Statistical Computing, https://www.r-project.org/) and GNU image manipulation software (GIMP, version 2.10.22, https://www.gimp.org/).

There was a skewed distribution in most WMH parcels (Fig. 2B). Among the 108 participants (Additional file 1: see Supplementary Table 1) in which we could evaluate the presence of WMH, WMH were more abundant around the lateral ventricles for all lobar regions except the occipital lobe, in which most WMH were found in layer 4 (Fig. 2A,B). Moreover, WMH burden between hemispheres tended to be symmetric for all WMH parcellations. Figure 2C shows the median WMH volume in the bullseye representation for the whole sample. WMH were more prevalent in parietal, frontal and occipital lobes. By contrast, temporal and BG WMH were less frequent, except in the layer closest to the ventricles (Layer 1).

We next evaluated the effect of age on WMH distribution by splitting the sample into tertiles (younger than 65 [n = 35, 32.4%], 65–70 [n = 29, 26.9%] and older than 70 [n = 44, 40.7%]). We then conducted pair-wise comparisons of WMH volume in each parcellation. As is shown in supplemental Fig. 2 (Additional file 1), participants in the second and third tertiles showed higher WMH burden in the parietal, temporal and occipital lobes than did participants in the first tertile, especially in distant layers. Moreover, participants in the third tertile additionally presented with higher WMH volume in frontal and BG regions as compared to younger (first tertile) participants. By contrast, differences between participants in the second and third tertiles were less frequent, indicating some degree of similarity between these age groups.

### Association between WMH parcellation and cognitive domains

**Figure 3:**
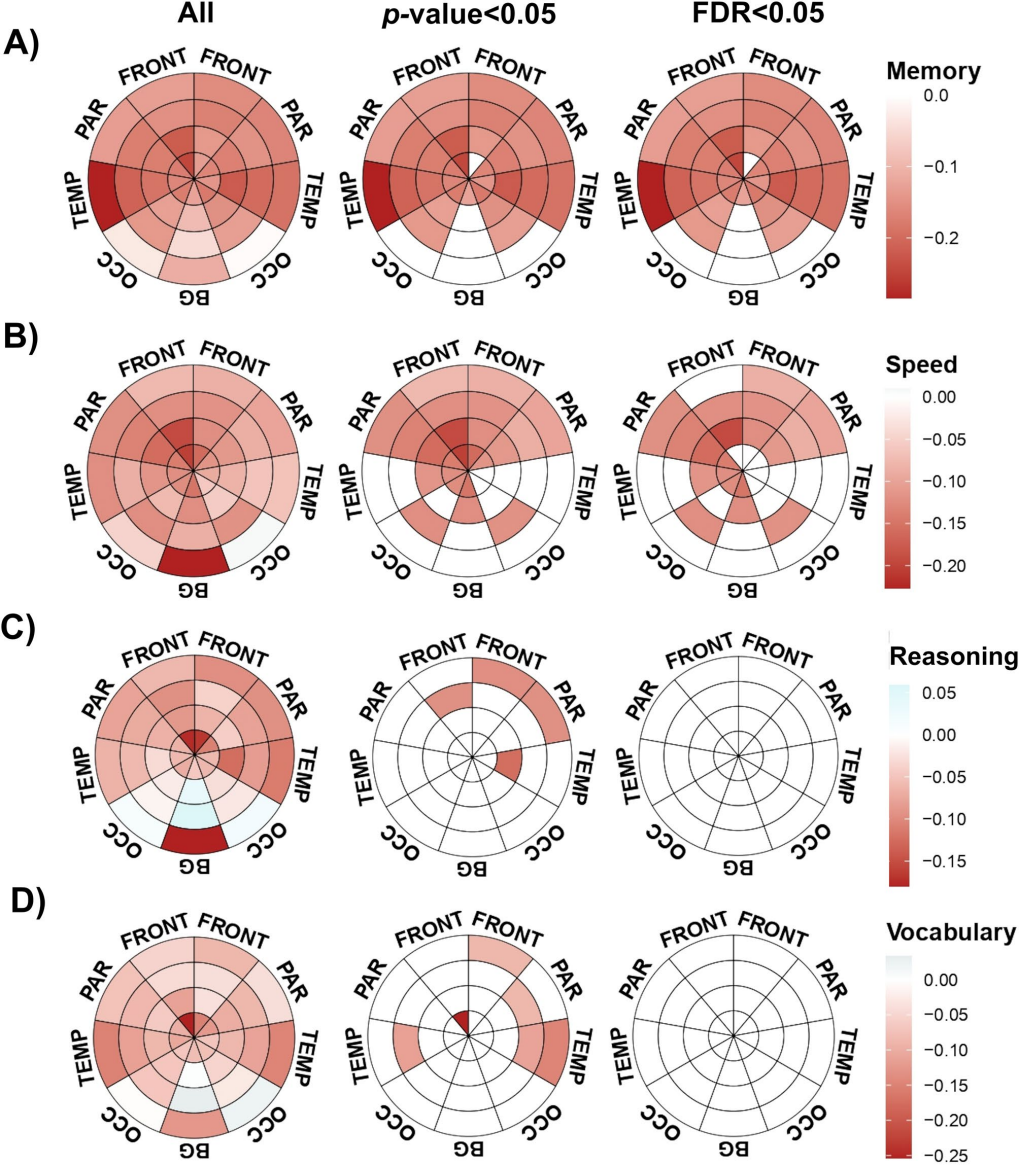
Effect of WMH distribution on cognitive domain: episodic memory (row A, N = 79), speed of processing (row B, N = 77), fluid reasoning (row C, N = 81) and vocabulary (row D, N = 77) were introduced in multiple linear regression models as dependent variables. Log transformed volumes for each parcellation were considered our predictors of interest, which were entered as independent variables in separate models (1 model per region), and adjusting for the effect of age, sex, education, BPF and hypertension. Colors represent the beta coefficients corresponding to the association between WMH volumes and cognition (red indicates negative correlations). First column represents the direction of the associations, independently of significance level. Second and third columns show only those coefficients having *p* or *q-*values lower than 0.05. This figure was constructed using ‘ggplot’ library (version 3.3.2, https://ggplot2.tidyverse.org/) included in R software (R version 3.6.3, 2020-02-29; 2020 The R Foundation for Statistical Computing, https://www.r-project.org/) and GNU image manipulation software (GIMP, version 2.10.22, https://www.gimp.org/).

We next explored the relationship between WMH parcellation and performance across the four cognitive domains (see Fig. 3). After correcting for multiple comparisons and adjusting for the effect of potential confounders (age, sex, education, BPF and hypertension), we observed that episodic memory performance was negatively associated with WMH burden in almost all regions of the bullseye representation (Fig. 3A). Interestingly, temporal WMH showed the strongest β coefficients as compared to other brain regions, especially in layer 4. By contrast, speed of processing was associated with WMH in frontal and parietal regions (Fig. 3B). Finally, after applying FDR correction, we observed no significant associations between WMH distribution and either fluid reasoning or vocabulary. As such, subsequent analyses are restricted to the relationship between WMH and episodic memory and speed of processing.

### WMH components

**Figure 4:**
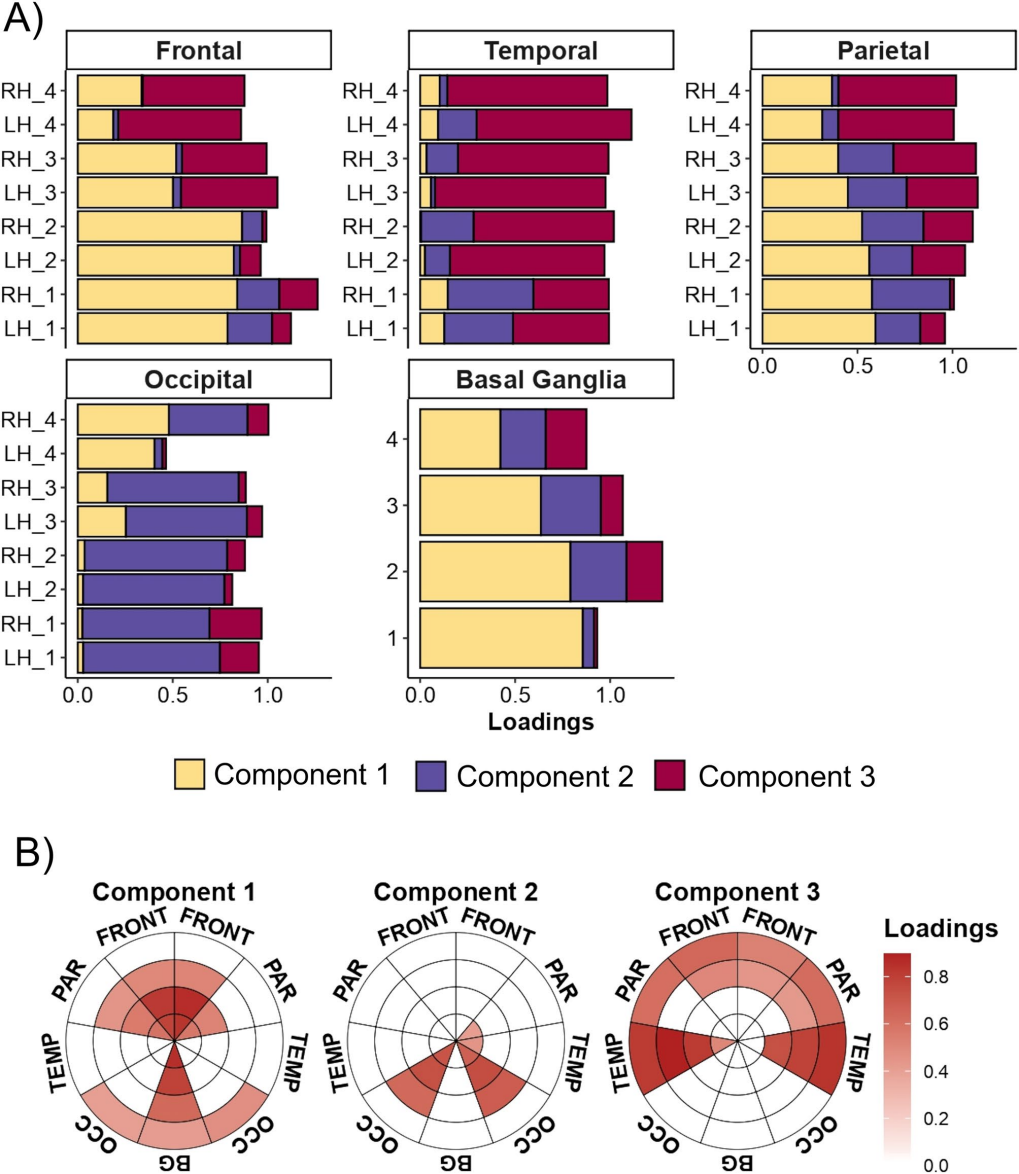
Principal component analysis (N = 108). In **(A)**, bars represent component loadings in each lobar region (frontal, temporal, parietal, occipital, and basal ganglia (BG)) by layers (1 to 4) and hemispheres (right and left). These loadings can be interpreted as the correlation coefficient between each pair of components and regions. In **(B)**, loadings higher than 0.4 were plotted against the bullseye map for each component. These second panel indicates those regions which show the highest correlation with each component. This figure was constructed using ‘ggplot’ library (version 3.3.2, https://ggplot2.tidyverse.org/) included in R software (R version 3.6.3, 2020-02-29; 2020 The R Foundation for Statistical Computing, https://www.r-project.org/) and GNU image manipulation software (GIMP, version 2.10.22, https://www.gimp.org/).

To provide clinical utility, we conducted a PCA on the full sample (n = 108). As is shown in supplementary Fig. 3 (Additional file 1), parallel analysis suggested that the 36 bullseye regions factored onto 3 PCs. After applying Oblimin rotation, we observed that the three dimensions explained 65% of the variance. Component loadings (the correlation matrix of each dimension with each of the 36 WMH sections) helped us to interpret each PC (Fig. 4A). We found that the first component largely explained the variance of WMH in frontal, parietal, and BG regions, especially within the periventricular region (layers 1 and 2), and to a lesser extent, in the deep region (layer 3). On the other hand, component 2 was mostly associated with WMH in the occipital lobe (especially from layers 1 to 3). Finally, component 3 explained variance from regions within the temporal lobe (layers 2 to 4), as well as juxtacortical regions from frontal and parietal lobes (layer 3 and 4). We labeled the three components (Fig. 4B) fronto-parietal periventricular WMH (explaining 26% of total variance), occipital WMH (15% of total variance), and temporal and juxtacortical WMH (24% of total variance). Supplementary Fig. 4 shows the neuroanatomical localization of these dimensions according to an example T1-weigthed image.

We then assessed the communality values of each WMH parcel in the PCA. As shown in supplementary Fig. 5 (Additional file 1), most variables presented communality values ranging from 0.6 to 0.8, indicating that the resulting components explained most of the variance of these features. However, occipital WMH in layer 4, as well as BG WMH from layers 3 to 4 presented communality values under 0.4, indicating that these variables had less in common with WMH in other regions and, thus, they were under-represented in the PCA.

### Association between WMH principal components and demographic factors and cognition

To understand the characteristics of the PCs, we first explored their association with age and sex (Additional file 1: supplementary Fig. 6). Age presented moderate positive correlations with the fronto-parietal periventricular (r_106_ = 0.41, *p* < 0.001) and the temporal and juxtacortical (rs_106_ = 0.37, *p* < 0.001) WMH PCs, and weak correlations with the occipital WMH component (r_106_ = 0.20, *p* = 0.039). Sex, on the other hand, was only significantly associated with occipital WMH, such that males had a higher mean occipital WMH burden than did females (x̄_males_ = 0.40 vs x̄_females_ = −0.33, *t*_105.3_ = 4.03, *p* < 0.001). As expected, components were positively correlated (Additional file 1: supplementary Fig. 6C), with the fronto-parietal periventricular/temporal and juxtacortical pair having the strongest correlation (rs_106_ = 0.66, *p* < 0.001). However, as shown in supplementary Fig. 7 (Additional file 1), we found a non-linear trend in the association between temporal and juxtacortical WMH and the other two components. Specifically, temporal and juxtacortical WMH exponentially increased with the presence of WMH in fronto-parietal periventricular and occipital areas, which indicates that those individuals with high WMH burden in temporal and juxtacortical regions were also those individuals who had advanced WMH burden in other regions.

Finally, we investigated which WMH components were independently associated with episodic memory (N = 79) and speed of processing (N = 77), after controlling for the effects of age, sex, education, BPF and hypertension (Table 1). We found that only temporal and juxtacortical WMH were independently associated with episodic memory performance (β = −0.34, bootstrapped 95% confidence interval = −0.60 to −0.12, *t* = −3.52, *p* ≤ 0.001). In contrast, only fronto-parietal periventricular WMH were associated with speed of processing performance after controlling for potential confounders (β = −0.23, bootstrapped 95% confidence interval = −0.37 to −0.05, *t* = −2.45, *p* = 0.017). In both models we observed variance inflation factors under 2, which indicates the absence of concerning multicollinearity.

**Table 1:** Multivariate regression models showing the relationship between WMH and cognitive domains after adjusting for potential confounders.

	Memory				Speed of processing			
	β (95% CI)	*t*-statistic	*p*-value	BS-95% CI	β (95% CI)	*t*-statistic	*p*-value	BS-95% CI
Fronto-parietal periventricular WMH	−0.03 (−0.21; 0.16)	−0.300	0.7651	−0.03 (−0.21; 0.17)	−0.23 (−0.42; −0.04)	−2.4519	0.0170	−0.23 (−0.37; −0.05)
Occipital WMH	−0.10 (−0.26; 0.07)	−1.1895	0.2386	−0.1 (−0.24; 0.04)	−0.05 (−0.21; 0.11)	−0.6739	0.5029	−0.05 (−0.24; 0.10)
Temporal & juxtacortical WMH	−0.34 (−0.53; −0.15)	−3.5234	< 0.001	−0.34 (−0.6; −0.12)	−0.06 (-0.25; 0.13)	−0.5916	0.5563	−0.06 (−0.2; 0.17)
Brain parenchymal fraction, SD	0.20 (0.02; 0.38)	2.1976	0.0316	0.20 (0.04; 0.33)	0.03 (−0.16; 0.22)	0.3555	0.7234	0.03 (−0.13; 0.18)
Age, years	0.03 (0.00; 0.05)	2.0331	0.0462	0.03 (0.00; 0.05)	−0.01 (−0.04; 0.02)	−0.7295	0.4685	−0.01 (−0.04; 0.02)
Education, years	0.08 (0.01; 0.15)	2.2565	0.0275	0.08 (0.01; 0.16)	0.06 (−0.01; 0.13)	1.6464	0.1047	0.06 (0.00; 0.14)
Sex, female	−0.04 (−0.39; 0.31)	−0.2449	0.8073	−0.04 (−0.37; 0.30)	−0.07 (−0.42; 0.29)	−0.374	0.7097	−0.07 (−0.44; 0.27)
Hypertension	−0.06 (−0.42; 0.30)	−0.3054	0.7611	−0.06 (−0.39; 0.29)	−0.03 (−0.39; 0.32)	−0.1781	0.8593	−0.03 (−0.35; 0.34)

Multiple linear regression models showing the relationship between WMH components and episodic memory and speed of processing after controlling for potential confounders (brain parenchymal fraction, age, education, sex and hypertension). Values indicate β estimates with 95% confidence intervals, *t*-statistic, *p*-values and bootstrapped confidence intervals.

*BS* bootstrap, *CI* confidence interval, *SD* standard deviation, *WMH* white matter hyperintensities.

## Discussion

In this manuscript, we assessed the distribution of WMH using a novel bullseye coordinate system proposed by Sudre et al. (2018), which segregates white matter into 36 parcels according to the lobar region and the distance from the ventricles^22^. This refined evaluation allowed us to investigate how different well-defined cognitive constructs map onto WMH distribution within the brain. We found that memory function was negatively correlated with WMH volume in almost all brain parcellations, while processing speed principally correlated with frontal WMH. Additionally, this classification allowed us to investigate how WMH in different brain regions grouped according to their correlation, finding three latent variables: fronto-parietal periventricular WMH (explaining principally WMH in frontal and parietal lobes, as well as in the BG, especially at the periventricular region); occipital WMH; and temporal and juxtacortical WMH (involving WMH in the temporal lobe, and at the juxtacortical region from frontal and parietal lobes). We found that whereas temporal and juxtacortical WMH were independently associated with episodic memory deficits, fronto-parietal periventricular WMH predicted worse performance in speed of processing tasks.

The majority of studies that have explored the impact of WMH on cognition have either considered the total WMH volume or have split WMH into PV- and D-WMH. Thus, it is challenging to compare our results with extant literature. However, Brugulat-Serrat and collaborators (2020), who evaluated WMH using the bullseye representation, also reported that juxtacortical WMH in frontal and parietal lobes were related to memory performance, although they did not report any association between memory and temporal WMH. Moreover, they found that speed of processing was associated with WMH at deep regions from the fronto-parietal area (concentric layers 3 and 4), as well as periventricular hyperintensities from the temporal region. Data from our sample is consistent with these past findings. In the current study, we found that speed of processing was mostly associated with the fronto-parietal region, a dimension that principally represented the periventricular region (layers 1 and 3). These results align with another study conducted in this cohort, in which parietal WMH mediated the effect of age on speed of processing^20^. However, our study additionally extends these past results by using the bullseye representation to reduce the dimensions to find latent constructs, which enabled us to investigate the independent contribution of each component to cognitive impairment.

This analysis revealed that temporal and juxtacortical WMH were independently associated with episodic memory function. Of note, dysexecutive function in vascular cognitive impairment is typically thought to arise because subcortical cerebrovascular burden principally interrupts fronto-subcortical circuits especially involved in executive functions and mood^45^, while memory is relatively preserved. However, it is important to consider that memory is a broad concept involving several sub-components such as episodic memory (recollection), familiarity and free-recall^46^, which may be influenced differentially by WMH. Our findings mirror those of Smith and collaborators (2011), who reported that temporal and occipital WMH were related to episodic memory impairments in a sample of both healthy older adults and patients with cognitive impairment^47^. Longitudinal data from Silbert et al. (2008) showed that D-WMH growth paralleled decline in episodic memory in a sample of older individuals without dementia. On the other hand, other measures of memory such as free-recall—which is thought to rely on frontal regions^48^—have been associated with PV-WMH, after controlling for D-WMH and other confounders^49^.

Therefore, episodic memory has been principally associated with WMH at the deep location, as well as in the temporal lobe or juxtacortical regions. WMH in these regions were less frequent in our study and were especially present in individuals with a higher WMH burden in the rest of the brain. Habes and colleagues (2018) recently showed that the increase in frontal PV-WMH precedes the accumulation of lesions at other locations, which might suggest that WMH progress from the periventricular to juxtacortical areas^50^. Given this, it is possible that the presence of WMH in the temporal lobe or juxtacortical areas, rather than being a specific pattern of distribution of WMH, might indicate that patients had a longer progression of cerebrovascular disease, probably accelerating Alzheimer’s disease (AD)-related pathology as amyloid beta accumulation (Aβ)^51,52^ or hippocampal atrophy^53^. Similarly, age-related decline in free-recall neuropsychological tests have been shown to precede impairment in episodic memory function^54^, while the latter is relative preserved until advanced stages of cognitive impairment^55^. If this is the case, PV-WMH would be predicted to show stronger associations with tests of executive functions or free-recall because these phenomena (both WMH and cognitive impairment) tend to appear early in the clinical continuum, while episodic memory impairment and progression of WMH to the juxtacortical or temporal regions might require longer clinical evolutions. Consequently, longitudinal studies that use the bullseye representation, coupled with cognitive tests that include both detailed clinical assessments and multiple measures of episodic memory and executive function, could help shed light on the progression of WMH and whether different patterns of WMH accumulation predict specific cognitive phenotypes.

In a previous analysis conducted in the same cohort, WMH were classified according to the brain lobe^20^. Using this criterion, WMH were not independently associated with memory performance and, in general, weaker associations between cognition and WMH were found. Hence, determining whether there are meaningful patterns of WMH distribution that can be derived from the bullseye parcellation has important clinical applications, as it might allow to identify which lesions are truly compromising the cognitive functioning. To our knowledge, only one other group to date has investigated the natural clusters of WMH using a data-driven approach like the one we employed^50^. Habes et al. (2018) found two clusters representing posterior WMH (principally including the occipital horns of lateral ventricles) and deep WMH (including lateral juxtacortical location). However, they also reported two additional clusters: frontal and dorsal WMH, while in our case these two regions were represented just by one unique cluster (fronto-parietal periventricular WMH). Although some differences may be due to distinct statistical procedures, our data, which revealed three primary clusters of WMH: fronto-parietal periventricular, temporal and juxtacortical, and occipital WMH, largely replicate those of Habes and colleagues.

Additionally, fronto-parietal periventricular WMH showed a stronger correlation with temporal and juxtacortical WMH as compared to occipital WMH. Similarly, distant layers from the occipital lobe were those regions that showed lowest communality values in the PCA, suggesting that these regions had less in common with WMH in the rest of the brain. This might be because white matter in the temporal region is principally irrigated by the perforating arteries arising from the middle cerebral artery, which also irrigates an important part of frontal and parietal lobes together with the anterior cerebral artery. On the other hand, occipital white matter is predominantly irrigated by the posterior cerebral artery, which might lead to differences in risk factors and hemodynamic characteristics as compared to the anterior circulation^56^. Interestingly, one study evaluating WMH according to the vascular territory also reported increased posterior WMH in males as compared to females^57^. The PCs that we report here thus might reflect both different stages of WMH progression, as well as differences in risk factors from specific vascular territories (fronto-parietal periventricular and temporal & juxtacortical WMH corresponded to lesions observed in vascular territories depending on the anterior and middle cerebral arteries, while occipital WMH are located mainly in regions irrigated by the posterior cerebral artery). These results provide motivation for future research to consider classification systems of WMH other than PV and DWMH, and data-driven approaches might be a useful tool to infer types of WMH.

Finally, our data revealed that age groups presented specific distributions of WMH. Participants in the second and third tertile of the age distribution in our sample presented with more WMH than subjects in the first tertile, especially in fronto-parietal and occipital regions. However, when we compared individuals in the second and third tertiles, we observed no significant differences. These findings may reflect a non-linear pattern of WMH progression, as proposed by van Leijsen et al. (2017), who reported that the progression of WMH especially accelerates during the sixth and seventh decades of life^58^.

## Strengths and limitations

This study has several limitations. First, the sample size was relatively small (N = 108), which may have reduced statistical power as well as the likelihood of observing other plausible associations between WMH patterns and cognitive deficits in fluid reasoning or vocabulary. Additionally, we did not evaluate other markers of subclinical cerebrovascular disease such as cerebral microbleeds, silent lacunar infarcts or enlarged perivascular spaces. As WMH progress in parallel to these lesions, it might be interesting to use the bullseye coordinate system to study these lesions as well. Finally, while we were able to adjust models for hypertension as measured through self-report, we did not have information about our participants’ blood pressure levels and, thus, we could not adjust our multivariate models for it. Further longitudinal studies including detailed clinical recordings will be of greater interest.

On the other hand, as strengths, we measured WMH using an automated model, bringing reliability to our measurements. Moreover, cognition was evaluated using a computer-based system, covering the most relevant cognitive functions. Finally, we used a data-driven approach to extract WMH clusters, giving insight into the natural aggrupation of WMH and granting novelty to our findings.

## Conclusions

In this manuscript we labeled WMH according to a novel classification system, which splits the brain white matter into 36 sections according to the lobar region and distance from the ventricles. We observed that WMH in frontal regions were correlated with performance in speed of processing, whereas the relationship between WMH and memory was more distributed throughout the brain, involving most of the 36 parcels. This coordinate system allowed us to study how WMH from different brain regions grouped according to their correlation. A PCA of the data suggested the existence of three latent variables representing fronto-parietal periventricular, occipital and temporal and juxtacortical WMH. Interestingly, fronto-parietal periventricular and temporal & juxtacortical WMH were independently associated with speed of processing and episodic memory, respectively. Hence, our results suggest that different spatial configurations of WMH might cause specific cognitive impairment phenotypes. The results presented here both extend current understanding of WMH contributions to cognition, and demonstrate the utility and benefit of classifying WMH using approaches that go beyond the traditional deep and periventricular WMH.

## Supplementary Information

Supplementary Information.

## Data Availability

This dataset is not publicly available, but data-sharing will be considered upon request of qualified researchers.

## Abbreviations

AD: Alzheimer’s disease
ANTs: Advanced normalization tools
Aβ: Amyloid-beta
BG: Basal ganglia
BIANCA: Brain Intensity AbNormality Classification Algorithm
BPF: Brain parenchymal fraction
DWMH: Deep white matter hyperintensities
eTIV: Estimated total intracranial volume
FDR: False discovery rate
k-NN: K-nearest neighbor
MCI: Mild cognitive impairment
PC: Principal component
PCA: Principal components analysis
PVWMH: Periventricular white matter hyperintensities
WMH: White matter hyperintensities
