# Supplementary material for: Effects of white matter hyperintensities distribution and clustering on late-life cognitive impairment
Source: Sci Rep. 2022 Feb 4;12:1955. doi: 10.1038/s41598-022-06019-8 (PMC8816933; doi:10.1038/s41598-022-06019-8)
Supplement: Supplementary file 1 — Supplementary Information. [file 41598_2022_6019_MOESM1_ESM.docx]

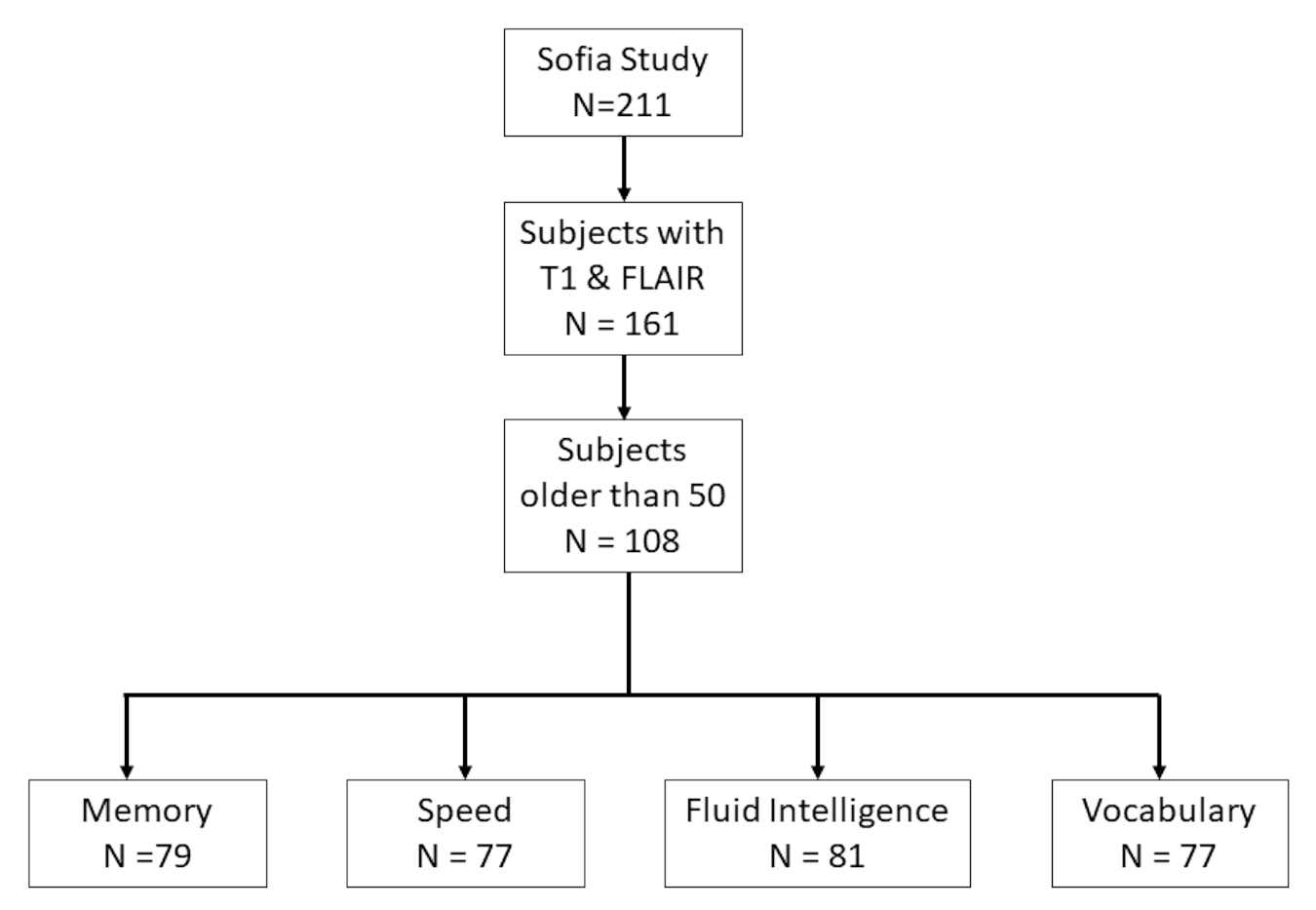


**Supplementary figure 1:** **Flow-chart of the sample.**

The figure was constructed using Microsoft Office Powerpoint 365 (version 2013, <https://www.microsoft.com/en-us/microsoft-365/powerpoint>) and GNU image manipulation software (GIMP, version 2.10.22, <https://www.gimp.org/>).

| **Supplementary Table 1:** **Principal characteristics of the sample** | |
| --- | --- |
| **Demographic and clinical Factors** | |
| **Age, years** | 69(64;73) |
| Number [58,65) | 35 (32.4%) |
| Number [65,71) | 29 (26.9%) |
| Number [71,84] | 44 (40.7%) |
| **Sex, Female** | 59 (54.6%) |
| **Education, years** | 15 (14;18) |
| **Hypertension** | 20 (20.2) |
| **BPF, percentage** | 72.5 (69.2;76.7) |
| **WMH Bullseye Parcellation** | |
| **Bilateral** |  |
| BG Layer 1, mm^3^ | 127.4 (26.5;296.1) |
| BG Layer 2 | 5.7 (0;29.7) |
| BG Layer 3 | 0 (0;2.9) |
| BG Layer 4 | 0 (0;0) |
| **Left Hemisphere** |  |
| Frontal Layer 1 | 319.9 (182.6;498.4) |
| Frontal Layer 2 | 214.8 (77.3;423.1) |
| Frontal Layer 3 | 32.1 (5.8;131.9) |
| Frontal Layer 4 | 22.3 (1.1;153.2) |
| Parietal Layer 1 | 115 (52.7;208.5) |
| Parietal Layer 2 | 122.3 (44.1;368.5) |
| Parietal Layer 3 | 71 (19.4;293.6) |
| Parietal Layer 4 | 18.7 (3.1;90.8) |
| Occipital Layer 1 | 11.4 (0.9;40.6) |
| Occipital Layer 2 | 12.7 (2.3;89.3) |
| Occipital Layer 3 | 35.8 (9.1;102) |
| Occipital Layer 4 | 173.2 (19.4;407.7) |
| Temporal Layer 1 | 1.8 (0;10.5) |
| Temporal Layer 2 | 0 (0;5.9) |
| Temporal Layer 3 | 0 (0;2.1) |
| Temporal Layer 4 | 0 (0;0) |
| **Right Hemisphere** |  |
| Frontal Layer 1 | 281 (176;463) |
| Frontal Layer 2 | 208.1 (79.4;526.7) |
| Frontal Layer 3 | 36.8 (4.8;177.8) |
| Frontal Layer 4 | 22.5 (0.9;160.3) |
| Parietal Layer 1 | 129.6 (63.2;227.2) |
| Parietal Layer 2 | 93 (29.3;358.3) |
| Parietal Layer 3 | 52 (6.2;299) |
| Parietal Layer 4 | 17.3 (1.7;91.9) |
| Occipital Layer 1 | 20 (3;52.6) |
| Occipital Layer 2 | 29.9 (4.7;76.8) |
| Occipital Layer 3 | 23 (2.4;87.7) |
| Occipital Layer 4 | 146.8 (17.2;314.5) |
| Temporal Layer 1 | 3.9 (0;17.2) |
| Temporal Layer 2 | 0 (0;7.1) |
| Temporal Layer 3 | 0 (0;5.2) |
| Temporal Layer 4 | 0 (0;0) |

Principal characateristics of the sample. Results are reported as median (interquartile range) or number (percentage). WMH volume adjusted by the intracranial cavity is expressed in mm^3^.

Keywords: BG, basal ganglia; BPF, brain parenchymal fraction.


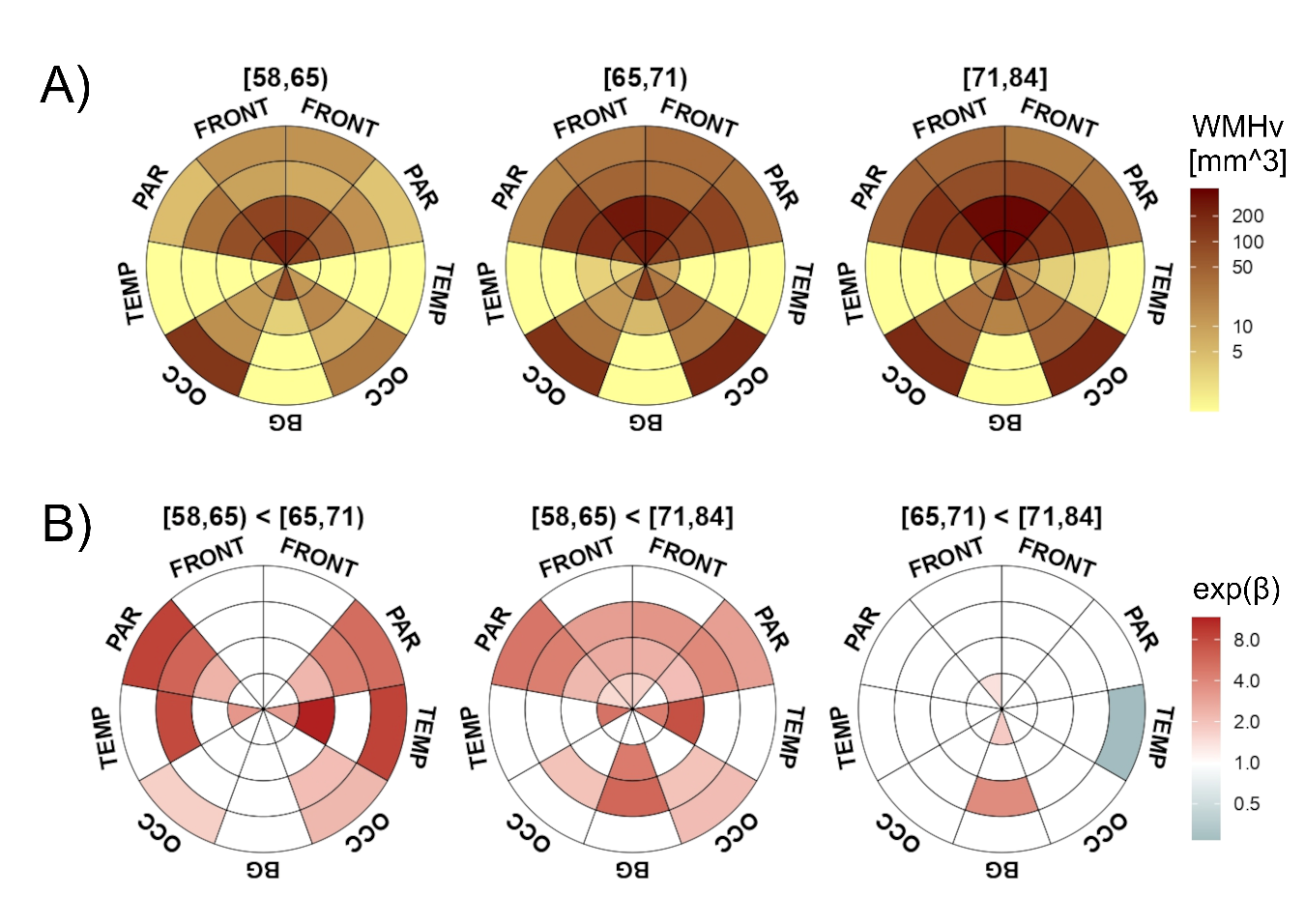


**Supplementary Figure 2:** **Effect of age on WMH distribution.** *Panel A:* Median WMH in each region by age group (age tertiles in the whole sample, N=108). Colors represent the burden of WMH in mm^3^. Darker colors indicate higher WMH loads. *Panel B:* WMH distribution was compared between groups using a general linear model assuming a gamma distribution with log link. Each Bullseye plot corresponds to a contrast (according to the label at the top of each plot). Colors represent the exponential function of β coefficients for each parcellation when *p*-value was lower than 0.05. Hence, values should be interpreted as the relative difference in WMH for each contrast. For example, a value of ‘2’ in a specific parcel of the fist plot means that [65, 71) individuals have double the WMH in that parcel as compared to [58, 65) individuals.

This figure was constructed using ‘ggplot’ library (version 3.3.2, <https://ggplot2.tidyverse.org/>) included in R software (R version 3.6.3, 2020-02-29; 2020 The R Foundation for Statistical Computing, <https://www.r-project.org/>) and GNU image manipulation software (GIMP, version 2.10.22, <https://www.gimp.org/>).


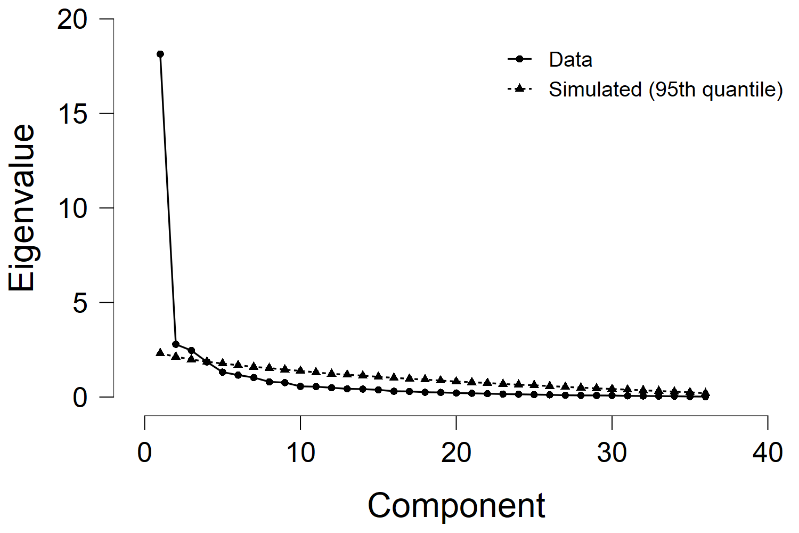


**Supplementary Figure 3:** **Scree plot of parallel analysis.** Triangles show eigenvalues corresponding to simulated data (95^th^ quantile), while circles show the actual principal components analysis scree plot (before rotation). Parallel analysis suggested the presence of 3 significant components, observed as the lack of overlap between simulated and real scree pots.

This figure was constructed using ‘ggplot’ library (version 3.3.2, <https://ggplot2.tidyverse.org/>) included in R software (R version 3.6.3, 2020-02-29; 2020 The R Foundation for Statistical Computing, <https://www.r-project.org/>) and GNU image manipulation software (GIMP, version 2.10.22, <https://www.gimp.org/>).


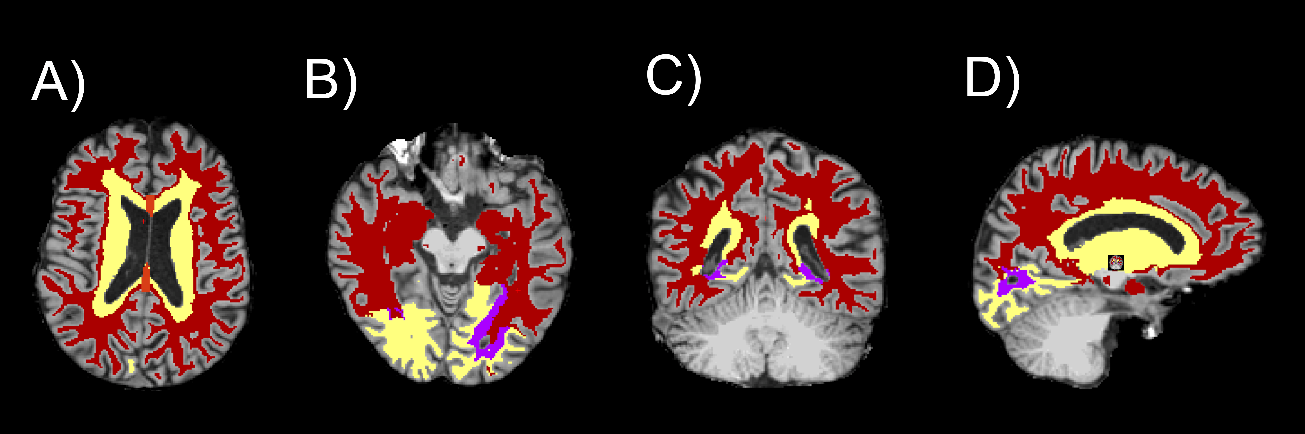


**Supplementary Figure 4:** **Neuroanatomical localization of each WMH component**. The fronto-parietal component (shown in yellow) corresponds to WMH surrounding the periventricular region from both the frontal and parietal lobes, and, to a lesser extent, distant regions from both frontal and occipital lobes. The temporal and juxtacortical component (shown in red) mainly correlates with WMH in juxtacortical regions and in the temporal area. Finally, occipital WMH (shown in purple) represents hyperintense lesions surrounding the periventricular area in the occipital lobe. Panels show these localizations in the axial (A and B), coronal (C) and sagittal (D) planes. Although regions are labeled with only one color in this figure, most white matter regions shared variance between components. For instance, juxtacortical regions from the frontal lobe shared variance between fronto-parietal and temporal-juxtacortical components. A more detailed explanation of these results can be found in Figure 4.

This figure was constructed using Freesurfer software (version 7.1., <https://surfer.nmr.mgh.harvard.edu/>) and GNU image manipulation software (GIMP, version 2.10.22, <https://www.gimp.org/>).


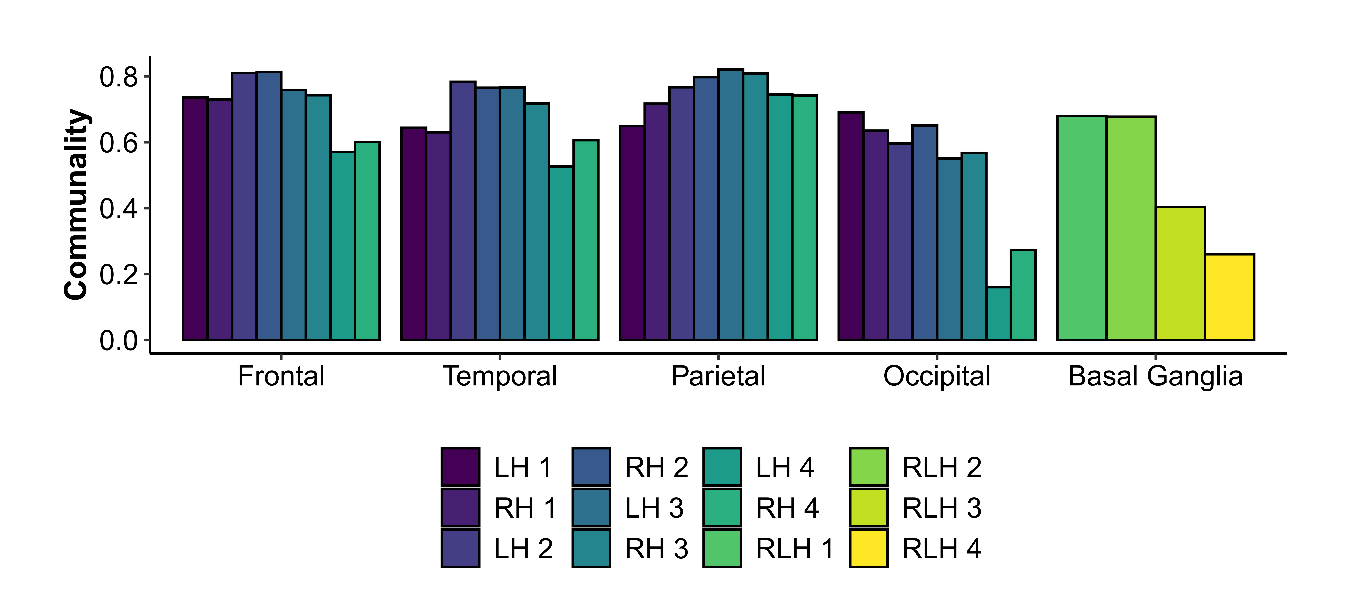


**Supplementary Figure 5:** **Communality values.** Communality values represent the sum of squares of weights of each variable in each component. A variable having a higher communality value indicates that the resulting components from the PCA explain a higher percentage of variance of this variable.

Key: LH, left hemisphere; RH, right hemisphere; RLH, right and left hemisphere.

This figure was constructed using ‘ggplot’ library (version 3.3.2, <https://ggplot2.tidyverse.org/>) included in R software (R version 3.6.3, 2020-02-29; 2020 The R Foundation for Statistical Computing, <https://www.r-project.org/>) and GNU image manipulation software (GIMP, version 2.10.22, <https://www.gimp.org/>).


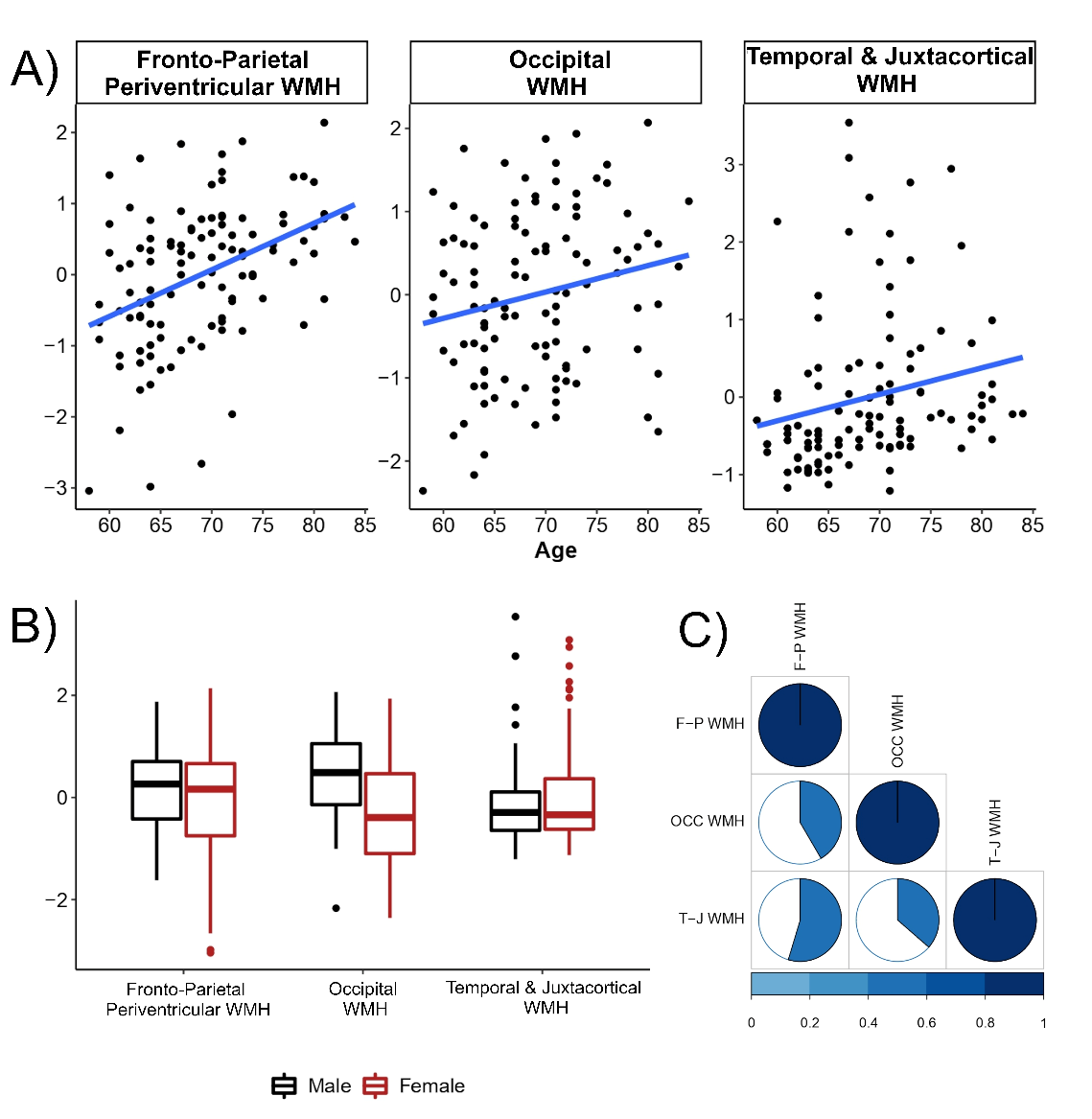


**Supplementary Figure 6.** **Components Characteristics.** *Panel A*: correlation between each component and age. *Panel B*: effect of sex on components. *Panel C*: correlation coefficients between components.

Key: F-P, fronto-parieta periventricular; OCC, occipital; T-J, temporal and juxtacortical; WMH, white matter hyperintensities.

This figure was constructed using ‘ggplot’ library (version 3.3.2, <https://ggplot2.tidyverse.org/>) included in R software (R version 3.6.3, 2020-02-29; 2020 The R Foundation for Statistical Computing, <https://www.r-project.org/>) and GNU image manipulation software (GIMP, version 2.10.22, <https://www.gimp.org/>).


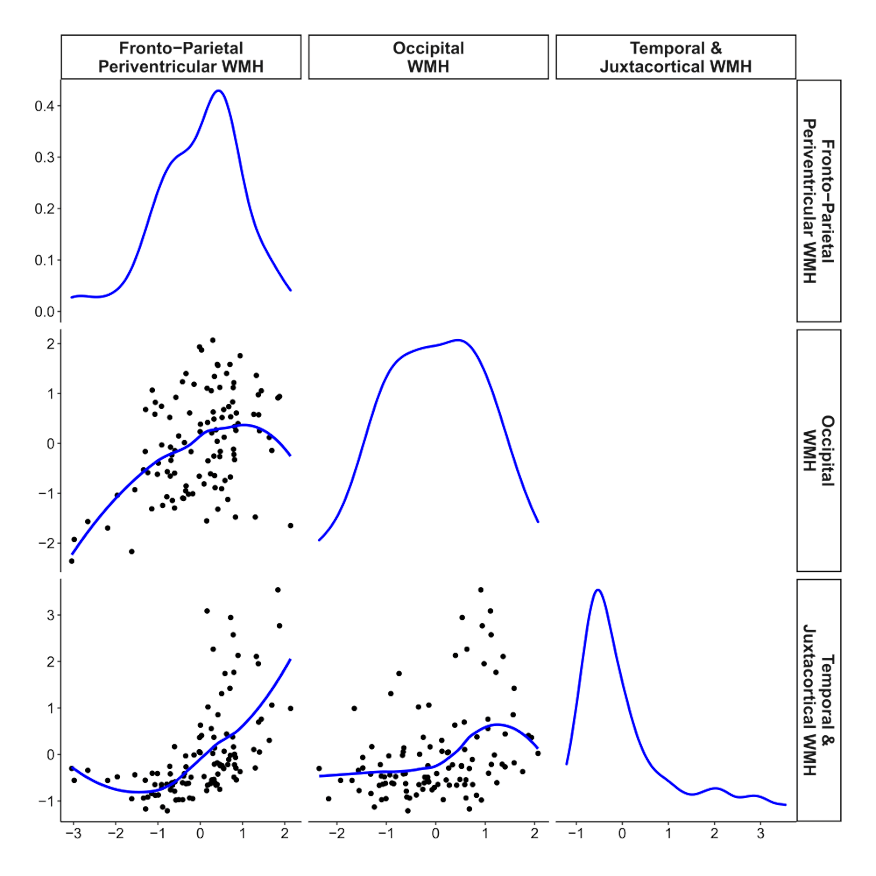


**Supplementary Figure 7.** **Relationship between WMH components.** Lower panels show the relationship between WMH components and the Loess (Locally Weighted Scatterplot Smoothing) fit in blue. Diagonal panels show the density function of each component.

Key: WMH, White matter hyperintensities.

This figure was constructed using ‘ggplot’ library (version 3.3.2, <https://ggplot2.tidyverse.org/>) included in R software (R version 3.6.3, 2020-02-29; 2020 The R Foundation for Statistical Computing, <https://www.r-project.org/>) and GNU image manipulation software (GIMP, version 2.10.22, <https://www.gimp.org/>).
